# Influence of Cavity Designs on Fracture Resistance: Analysis of the Role of Different Access Techniques to the Endodontic Cavity in the Onset of Fractures: Narrative Review

**DOI:** 10.1155/2024/1648011

**Published:** 2024-07-30

**Authors:** Mario Dioguardi, Davide La Notte, Diego Sovereto, Cristian Quarta, Angelo Martella, Andrea Ballini

**Affiliations:** ^1^ Department of Clinical and Experimental Medicine University of Foggia, Via Rovelli 50, Foggia 71122, Italy; ^2^ DataLab Department of Engineering for Innovation University of Salento, Lecce, Italy

## Abstract

**Objectives:**

In recent years, new endodontic access techniques have been proposed with the aim of preserving as much dental tissue as possible for subsequent prosthetic rehabilitation. It has indeed been demonstrated that the success of this therapy is essential and dependent on the proper execution of endodontic cavity access. The main objective is to provide a comprehensive and up-to-date overview of the new access techniques in endodontics in order to guide clinical practice toward a more precise and qualitative approach. As of today, there is still no universally standardized and recognized taxonomy for the different access cavity designs described in the literature. It has been observed that there are various nomenclatures in the literature. The cavity access designs described mainly in the literature can be categorized into six groups: Traditional Access Cavity (TradAC), Conservative Access Cavity (ConsAC), Ultra-Conservative Access Cavity (UltraAC), Truss Access Cavity (TrussAC), Caries-Guided Access Cavity (CariesAC), and Restoration-Guided Access Cavity (RestoAC).

**Materials and Methods:**

The drafting of this narrative review followed the indications of the SANRA (Scale for the Assessment of Narrative Review Articles). A search for scientific articles was conducted on the PubMed and SCOPUS databases, using the following search query: ((truss) OR (conservative) OR (ninja) OR (traditional)) AND access AND endodontic.

**Results:**

The initial search yielded a total of 941 articles. After removing duplicates using EndNote X8 software, the number of articles decreased to 785. By applying the inclusion and exclusion criteria, a total of 64 articles were obtained. Among these, 20 articles were finally selected for the purposes of this review, 11 literature reviews and 9 ex-vivo studies.

**Conclusion:**

Studies on fracture resistance have yielded heterogeneous results. For anterior teeth, studies do not find a significant relationship between different endodontic access cavities and fracture resistance. However, in the posterior sector, there is more discrepancy and many positive results for minimally invasive access cavities seem to relate to molars. Therefore, it can be concluded that the evidence supporting the influence of endodontic preparations on dental fracture resistance is still limited. Research on new endodontic access techniques holds significant clinical relevance in contemporary endodontics. The evolution of dental technologies, including cone beam computed tomography (CBCT) and computer-guided cavity preparation, has ushered in the era of minimally invasive endodontics. This shift aims to enhance the precision and quality of endodontic treatments while preserving maximum healthy dental tissue for subsequent prosthetic rehabilitation. The success of endodontic therapy is closely tied to the proper execution of access to the endodontium, influencing all phases of endodontic treatment and playing a role in determining fracture resistance for subsequent rehabilitation phases. The dichotomy between traditional and minimally invasive approaches has spurred clinical investigations. Specifically, within the scientific community, doubts have been raised about the potential limitations of minimally invasive access cavities. Concerns include their impact on canal orifice localization and raise questions about their influence on the overall success of endodontic treatment. This review holds clinical significance as it sheds light on the evolving landscape of endodontic access techniques, analyzing the anatomical trajectory, carefully examines the transition to minimally invasive approaches, and critically assesses existing scientific evidence and concerns surrounding these developments, contributing to an informed decision-making process in clinical practice.

## 1. Introduction

In recent years, new techniques for endodontic access have been proposed, with the aim of improving the precision, quality, and effectiveness of endodontic treatment while preserving as much dental tissue as possible for subsequent prosthetic rehabilitation.

It has been demonstrated that the success of this therapy is inseparable from the proper execution of a correct access to the endodontium, which can influence all subsequent phases of canal treatment: localization of canal orifices, canal shaping and disinfection, obturation, and the achievement of a biological seal [1], as well as the exposure of the subsequent prosthetic restoration to fractures [2].

For these reasons, we have decided to examine and evaluate the new techniques for endodontic access through an analysis of the scientific literature on the subject. The theoretical principles underlying these techniques will be presented, as well as the results of scientific studies that have assessed - or refuted - their effectiveness. The main objective is, indeed, to provide a comprehensive and up-to-date overview of the new techniques for endodontic access in order to guide clinical practice towards a more precise and qualitative approach.

One of the first descriptions of the traditional cavity design was given in 1920 by Crane et al. [3] in which the author asserted that such a cavity should provide direct access to each canal in line with its longitudinal axis, while also attempting to preserve the walls of the pulp chamber. However, the designs of traditional access cavities were better described only in 1965 by Ingle [4], where a sequence of steps was first presented for the creation of such accesses. In particular, the author adapted the concepts of cavity preparation previously described by Black [5], which showed a sequence of procedures to follow.

Specifically, for the first time, attention was focused on two particular parameters: the convenience form and the extent of the endodontic opening for prevention [6]. The principle of “convenience form” suggested providing access as direct as possible to the orifice of the root canal (now commonly called “straight-line access”), achieved by extending the cavity walls more, while the extent for prevention, proposed by Ingle [7], aimed to widen the root canal in all its dimensions to achieve the convenience form to make all subsequent phases of endodontic treatment more efficient and, at the same time, to prevent complications.

Therefore, very generally, the principles of traditional access cavity preparation can be summarized in the following three key points:

1. Complete destruction of the roof of the pulp chamber, exposing the pulp horns [8].
2. Creation of a smooth and obstacle-free path to the canal orifices [9].
3. Preservation of healthy dental structure [10].

However, due to technical limitations represented by endodontic instruments, the design of traditional endodontic access cavities has remained virtually unchanged for decades, following the precepts previously described: straight-line access to root canals and divergent walls to facilitate the localization of canal orifices and to avoid complications in subsequent phases of canal treatment, such as the fracture of canal instruments and the failure to locate root canals [11, 12].

However, the marketing of new dental technologies in the last two decades, such as CBCT (cone beam computed tomography) and the possibility of computer-guided cavity preparation, has opened up the possibility of creating minimally invasive access cavities, with the aim of preserving as much healthy dental tissue as possible.

We know that minimal invasiveness is a concept that has already firmly established itself in other areas of dentistry, focusing on the maximum preservation of dentin, particularly in endodontics. The theoretical basis for this concept was provided by two publications by Clark and Khademi [13, 14], in which the authors focused on the impact of the traditional access cavity (complete destruction of the roof of the pulp chamber and straight-line access to the canal orifices) on the long-term prognosis of teeth undergoing endodontic treatment. Alternatively, for the first time, a cavity design was proposed that aimed to preserve as much as possible the roof of the pulp chamber, as well as what they called “pericervical dentin,” a portion of dentin located approximately 4 mm above and 6 mm below the alveolar crest, which they considered crucial in transmitting occlusal forces to the dental root [15, 16].

The same concept was then applied to anterior teeth, in which it is more precisely referred to as “pericervical dentin,” as there is a strong concentration of tensile forces at the cingulum on incisors during their masticatory function. Therefore, it was deduced that, in the same way, the removal of pericervical dentin in anterior teeth would result in less resistance to fracture. To preserve these structures, it was considered appropriate to partially preserve the roof of the pulp chamber, which would reduce cusp deflection by occlusal forces, once the dental element was prosthetically restored. In fact, teeth subjected to endodontic treatment have a lower long-term survival rate compared with intact teeth, precisely because of reduced resistance to fracture [17].

Because of this, over the years, many clinical studies have attempted to analyze how endodontic treatment represents a risk factor for dental fracture, following the removal of dental tissue for caries removal and, indeed, for the preparation of the endodontic access cavity, as well as for shaping in the subsequent stages of canal treatment [18].

Therefore, much attention must be paid to the fact that these theories and concepts of Clark et al. [13, 14] were not presented with the support of scientific evidence at the time of publication. Therefore, we have decided to conduct this literature review to critically and objectively analyze the current status in this regard. Indeed, while it is undeniable that, in recent years, the concept of minimally invasive endodontics is gaining greater acceptance within clinical dentistry and it should be remembered that there is limited scientific evidence in support of this concept [19].

On the contrary, only in recent years has the scientific community begun to express doubts about these minimally invasive access cavities, as they could constitute a limitation for the stages of canal orifice localization [20], irrigation [21], shaping [22], and root canal filling [23] and thus for the entire endodontic treatment[24].

Moreover, it should not be underestimated that access cavities of insufficient size could even lead to a higher incidence of accidents and complications during the endodontic treatment. This aspect will constitute the second objective of this literature review, to analyze, assuming that minimally invasive accesses bring benefits in terms of fracture resistance, how and to what extent they influence the subsequent stages of endodontic treatment, and thus its overall success [25].

In this regard, it should be noted that there is still no universally recognized and standardized taxonomy for the high number of access cavity designs. It has been found that there are numerous nomenclatures in the literature, with terms and acronyms that often overlap or, conversely, resemble each other while not referring to the same cavity design. For greater clarity and to exclude methodological biases, in this review, we have decided to adopt the nomenclature proposed by Silva et al. in 2020 [26]. In this article, the authors categorized cavity designs into six groups: Traditional Access Cavity (TradAC), Conservative Access Cavity (ConsAC), Ultra-Conservative Access Cavity (UltraAC), Truss Access Cavity (TrussAC), Caries-Guided Access Cavity (CariesAC), and Restoration-Guided Access Cavity (RestoAC)[27]. A brief description of each of these cavity designs, according to the proposal of the aforementioned authors, is provided [28].

1. Traditional Access Cavity (TradAC): In posterior teeth, it consists of complete removal of the roof of the pulp chamber, followed by achieving a straight-line access to the canal orifices, with slightly convergent axial walls, so that all orifices are visible within the opening. In anterior teeth, perpendicular access is obtained by removing the roof of the pulp chamber, pulp horns, the lingual portion of dentin, and further extending the access cavity to the incisal edge.
2. Conservative Access Cavity (ConsAC): The purpose of this opening is to minimize the destruction of dental tissue by preserving part of the roof of the pulp chamber. In posterior teeth, the preparation typically begins from the central pit of the occlusal surface and extends only as far as necessary to locate the canal orifices, with slightly converging axial walls towards the occlusal surface, thus preserving part of the roof of the pulp chamber [29].

This type of access can also be done with divergent walls. In anterior teeth, this access involves shifting the entry point from the cingulum to the incisal edge, on the lingual or palatal surface, creating a small triangular or oval-shaped cavity while preserving pulp horns and as much pericervical dentin as possible.

The philosophy behind ConsAC is to preserve part of the roof of the pulp chamber to distribute occlusal forces before they reach the pulp chamber floor, where fracture lines often develop in teeth after endodontic treatment [28].

1. Ultra-Conservative Access Cavity (UltraAC): Known as the “ninja' access, this cavity starts with the same approach described in the conservative technique but without further extensions, while preserving as much of the pulp chamber roof as possible. In anterior teeth, if the lingual surface of the crown is particularly concave, access can be made in the centre of the incisal margin, parallel to the longitudinal axis of the tooth [30].
2. Truss Access Cavity (TrussAC): It aims to preserve the dentin bridge between two (or more) small cavities made on the occlusal surface of the multirooted tooth to directly access the canal orifices of each root of a multirooted tooth. In mandibular molars, for example, two or three individual cavities can be created to access the mesial and distal canals, thus preserving the enamel and dentin between the different accesses [31].
3. Caries-Guided Access Cavity (CariesAC): Access to the pulp chamber is achieved by removing caries while preserving all remaining dental structures, including the pulp chamber roof [32].
4. Restoration-Guided Access Cavity (RestoAC): In restored teeth free of caries, access to the pulp chamber is obtained by removing existing restorations either completely or partially while preserving all remaining dental structures [33].

In this literature review, only the first four cavity designs, specifically TradAC, ConsAC, UltraAC, and TrussAC, will be examined. The UltraAC and TrussAC are often referred to as MiniAC (minimally invasive endodontic accesses). CariesAC and RestoAC, which represent nonstandardized and nonprotocolized access cavities, will be excluded as they, for the time being, represent the ideal approach in terms of maximum preservation of dental tissue.

The objective is to examine the impact of endodontic access techniques on fracture resistance through a critical analysis of the available scientific literature, analyzing the possible implications of access techniques on the structural integrity of the tooth. While providing an updated and comprehensive overview of the various endodontic access techniques, we identify the advantages, disadvantages, and clinical implications of each issue.

## 2. Materials and Methods

The drafting of this narrative review followed the indications of the SANRA (Scale for the Assessment of Narrative Review Articles) [34]. A checklist was also performed, taking into consideration the SANRA [34] and the guidelines provided by Green et al. [35] on the writing of a narrative literature review. The checklist is available in the supplementary materials (available here).

The validation of the research instrument involved several phases to ensure its robustness and reliability which concern expert consultation and protocol drafting, initial research, peer review, consistency checks, and the checklist.

### 2.1. Expert Consultation

The initial framework of the tool was reviewed and refined by consulting experts. This included feedback from senior researchers and academics experienced in systematic and narrative reviews. Furthermore, a research protocol was drawn up and was not registered on PROSPERO because the latter does not provide for registrations of in vitro studies and more generally narrative or scoping reviews.

Initial search: an initial search was conducted using certain keywords on certain databases (PubMed) to evaluate the effectiveness and completeness of the tool. Based on the results, further changes were made to the protocol with the addition of databases and keywords to improve the search and selection of the studies.

Peer review: the research protocol and the selection of records and more generally the research instrument was subjected to peer review within the authors' team, ensuring that all criteria and methods were critically evaluated and agreed upon.

### 2.2. Consistency Checks

Throughout the review process, consistency checks were performed to ensure that the protocol was applied consistently by all reviewers.

The students (D.L.D. and D.S.) who participated in the data selection and extraction process like the entire research group were involved in the validation process by refining the research techniques and a self-evaluation process of the preliminary results was conducted by them and by the group to identify possible strategies to improve the search for records.

The task of the investigators, and more specifically the reviewers, was as follows: A.B., in preliminary coordination with the research team, selected two reviewers (D.L.N. and D.S.) and a third reviewer (M.D.) as a supervisor who, in the event of a conflict regarding the studies to be included, would make the final decision on which studies to include.

The two reviewers (D.L.N. and D.S.), in collaboration with M.D. and A.B., were responsible for, after defining the outcomes and the research question with the group, selecting the databases and keywords used, as well as determining the inclusion and exclusion criteria and choosing the data to extract along with the synthesis methods (Tables). The drafting of the protocol and the decision not to register were the responsibility of the working group.

The identification of records and selection of studies via databases, with manual or software-based duplicate removal (EndNote 8.0), was carried out independently by the two reviewers (D.L.N. and D.S.). The subsequent review of the selected studies and the decision on the studies to include were conducted under the supervision of M.D.

D.L.N. and D.S. also independently extracted the data into tables, with a subsequent comparison of the data to minimize the risk of errors in reporting information. At this stage, A.M. verified the correct inclusion and reporting of biometric data of patients from the included studies.

This review analyzes and compares, as previously mentioned, the first four techniques, namely,

1. Traditional Access Cavity (TradAC)
2. Conservative Access Cavity (ConsAC)
3. Ultra-Conservative Access Cavity or “ninja” (UltraAC)
4. Truss Access Cavity (TrussAC)

It is worth noting that the last two access techniques, UltraAC and TrussAC, are often grouped under the broader category of minimally invasive access techniques (MiniAC).

Guided techniques, on the other hand, will be excluded. These involve the creation of software-guided templates after performing a CBCT scan, which already provide the correct angulation and extension for the access opening, significantly reducing the operator's experience's impact on the predictability of the procedure.

Guided endodontic access procedures can be further categorized into static and dynamic guided navigation, but they will not be examined, as their indication generally requires specific criteria (calcified canals, absence of canals with significant curvature), that do not overlap with the broader criteria for performing TradAC, ConsAC, UltraAC, or TrussAC.

The objectives of review are, therefore, to evaluate, based on the literature, the advantages and disadvantages of one access technique compared to another. This evaluation encompasses not only the prognostic perspective, i.e., how they influence each of the subsequent stages of endodontic treatment (ease of access and instrumentation of the root canals), but also the prevention of postendodontic complications, such as dental fractures.

A search for scientific articles was conducted on the PubMed database using the following search query: (truss) OR (conservative) OR (ninja) OR (traditional) AND access AND endodontic.

(“trusses”[MeSH Terms] OR “trusses”[All Fields] OR “truss”[All Fields] OR (“conservancies”[All Fields] OR “conservancy”[All Fields] OR “conservancy s”[All Fields] OR “conservation”[All Fields] OR “conservational”[All Fields] OR “conservations”[All Fields] OR “conservative”[All Fields] OR “conservatively”[All Fields] OR “conservatives”[All Fields] OR “conserve”[All Fields] OR “conserved”[All Fields] OR “conserves”[All Fields] OR “conserving”[All Fields]) OR “ninja”[All Fields] OR (“tradition”[All Fields] OR “tradition s”[All Fields] OR “traditional”[All Fields] OR “traditionals”[All Fields] OR “traditions”[All Fields])) AND (“access”[All Fields] OR “accessed”[All Fields] OR “accesses”[All Fields] OR “accessibilities”[All Fields] OR “accessibility”[All Fields] OR “accessible”[All Fields] OR “accessing”[All Fields]) AND (“endodontal”[All Fields] OR “endodontic”[All Fields] OR “endodontical”[All Fields] OR “endodontically”[All Fields] OR “endodontics”[MeSH Terms] OR “endodontics”[All Fields])

### 2.3. Translations

Truss: “trusses”[MeSH Terms] OR “trusses”[All Fields] OR “truss”[All Fields].

Conservative: “conservancies”[All Fields] OR “conservancy”[All Fields] OR “conservancy's”[All Fields] OR “conservation”[All Fields] OR “conservational”[All Fields] OR “conservations”[All Fields] OR “conservative”[All Fields] OR “conservatively”[All Fields] OR “conservatives”[All Fields] OR “conserve”[All Fields] OR “conserved”[All Fields] OR “conserves”[All Fields] OR “conserving”[All Fields].

Traditional: “tradition”[All Fields] OR “tradition's”[All Fields] OR “traditional”[All Fields] OR “traditionals”[All Fields] OR “traditions”[All Fields].

Access: “access”[All Fields] OR “accessed”[All Fields] OR “accesses”[All Fields] OR “accessibilities”[All Fields] OR “accessibility”[All Fields] OR “accessible”[All Fields] OR “accessing”[All Fields].

Endodontic: “endodontal”[All Fields] OR “endodontic”[All Fields] OR “endodontical”[All Fields] OR “endodontically”[All Fields] OR “endodontics”[MeSH Terms] OR “endodontics”[All Fields].

In addition, a search for articles on Scopus was also done using the following terms and search within the title abstract and keywords: (truss) OR (conservative) OR (ninja) OR (traditional) AND access AND endodontic.

### 2.4. Inclusion Criteria

1. Studies comparing fracture resistance between different endodontic access techniques (TradAC, ConsAC, UltraAC, TrussAC)
2. Studies comparing the prognostic outcome of different endodontic access techniques (TradAC, ConsAC, UltraAC, TrussAC)
3. *In vitro* studies conducted on human permanent teeth.

### 2.5. Exclusion Criteria

1. Studies conducted on resin or other artificial teeth
2. Studies conducted on animal teeth
3. Studies focusing on guided endodontic access cavities

Subsequently, the various articles were grouped based on their specific focus: fracture resistance or prognosis of endodontic treatment (if both topics were not addressed), to facilitate a more straightforward and comparable analysis of the results. Articles were then selected for each area of study (fracture resistance and endodontic prognosis), excluding those with lower scientific value that merely reiterated widely supported findings from high-value scientific articles. Conversely, articles of intermediate scientific value were considered for inclusion in cases where results were ambiguous or controversial, while still accounting for their varying levels of scientific relevance.

## 3. Results

The initial search, based on the input provided above without applying any filters, yielded a total of 941 articles. After removing duplicates using EndNote X8 software, the number of articles decreased to 785.

By applying inclusion and exclusion criteria based on the title, abstract, or keywords of the results, a total of 64 articles were identified. Among these studies, some investigated the prognosis of endodontic treatments performed on various access cavities, albeit in conjunction with different mechanical instrumentation systems. Similarly, investigations into fracture resistance involved comparisons of different resin composites for postendodontic reconstruction, albeit with variations in the upstream access cavities being considered.

Given the abundance of studies, it was deemed appropriate to exclude articles considered of lower qualitative significance. Among these, 20 articles were finally selected for the purposes of this review, 11 literature reviews and 9 ex-vivo studies.

1. Reviews: Shabbir et al. [36], Silva et al. [26] Ballester et al. [27] Saeed et al. [37], Silva et al. [12], Mandil et al. [38], Silva et al. [39], Kapetanaki et al. [40], Motiwala et al. [41], Chan et al. [42], and Maqbool et al. [43];
2. Studies: Patil et al. [44], Mowlood et al. [45], Saberi et al. [46], Makati et al. [47], Maske et al. [48], Özyürek et al. [49], Barbosa et al. [50], Rover et al. [51], and Plotino et al. [52].

Each of these articles analyzed the traditional access cavity (TradAC), which was compared in 12 articles with conservative access (ConsAC), in 13 articles with ultra-conservative access (UltraAC), and in 7 articles with the Truss cavity (TrussAC).

It should be noted that both systematic and narrative reviews and ex-vivo studies were included in this literature review. Among the latter, studies that compared the influence of the access cavity on fracture resistance were selected.

With regard to the parameters analyzed, 14 of the articles examined specifically evaluated the fracture resistance of different access cavities, while 4 articles compared the influence of the cavity design on aspects relating to the efficacy and prognosis of root canal treatment and 2 reviews relating to classification.

**Figure 1:**
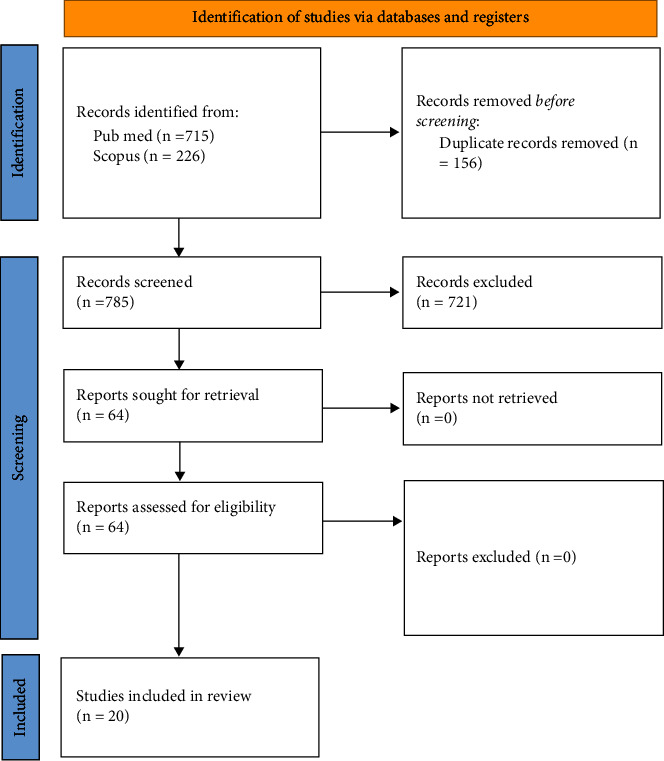
The flow chart of the different phases of the study selection process.

The entire selection process is widely described in the flow chart (Figure 1).

The main data extracted have been reported in 2 separate tables.  Table 1 shows the data relating to the narrative and systematic reviews, while Table 2 shows the data relating to the ex-vivo studies.

The average sample size of the studies included in the review ranges from Plotino et al. [52], with 160 samples, to the study by Barbosa et al. [50], with 30 samples. The mean sample size was 63.77 with a standard deviation (SD) of ± 35.558. Meanwhile, the total number of teeth included in the ex-vivo study review was approximately 574.

## 4. Discussion

Based on the results of the 20 articles examined, it becomes evident that a well-designed endodontic access cavity is crucial for the ultimate prognosis of root canal treatment, both in the medium and long term.

Several authors have conducted studies to compare traditional access cavities (TradAC), conservative access cavities (ConsAC), ultra-conservative access cavities (UltraAC), and Truss access cavities (TrussAC) with each other. This comparison extends not only to the subsequent fracture resistance of the tooth but also to the instrumentation, disinfection, and obturation processes since each of these steps depends on the configuration of the access cavity.

In line with the objectives set in this literature review, greater attention has been focused on the resistance of teeth undergoing root canal treatment, as it represents the main topic of debate concerning the various access cavities. In recent years, the creation of smaller access cavities has been considered to address these issues. The underlying assumption is that the less dental tissue is removed, the greater the resistance to dental fracture.

### 4.1. Resistance of Fracture

The main topic of discussion revolves around the relationship between access cavities and the resulting fracture resistance of endodontically treated teeth. This is indeed the reason why minimally invasive openings were proposed just over a decade ago, with the aim of reducing this risk.

It is well known that the most common consequence of such fractures is the extraction of the dental element.

In particular, the foundation of these proposals is based on the assumption that the fracture resistance of teeth is correlated with the preservation or removal of dental tissue, both in terms of creating the access cavity and shaping the root canal. Therefore, more conservative cavities would result in greater preservation of the volume of dental tissue, leading to increased long-term resistance of the element [13].

The spread of minimally invasive access cavities is grounded in the philosophy that pericervical dentin plays a crucial role in fractures of endodontically treated teeth. This is because the location of these fractures primarily concentrates on the level of the pulp chamber floor. It follows that the more we can limit mechanical stress on pericervical dentin, the lower the risk of postendodontic treatment fractures for the dental element.

Hence, the initial step of this systematic review was to verify whether different designs of endodontic access cavities are indeed associated with greater or lesser loss of dental tissue, and more specifically concerning this pericervical dentin, which is considered crucial for the long-term prognosis of endodontically treated teeth.

In this regard, the analyzed studies yielded consistent and unambiguous results, confirming that the Traditional Access Cavity (TradAC) represents the access cavity design with the most substantial destruction of dental tissue [36].

However, it is not yet certain how the design of the access cavity influences the mechanical stresses experienced by healthy dental tissue, both quantitatively and qualitatively [36].

Specifically, in an *in vitro* study involving 60 molars, a comparison was made between the thickness of the remaining dentin and the fracture resistance of two groups of samples. One group was prepared with the Traditional Access Cavity (TradAC), while the other was prepared with the Conservative Access Cavity (ConsAC), retaining part of the peripheral portion of the pulp chamber roof.

To confirm how these cavity designs resulted in different removals of pericervical dentin, the residual dentin thickness of each tooth was measured using CBCT, before and after the execution of the access cavity. In this manner, a statistically significant difference in dentin thickness between the two groups was confirmed, with ConsAC preserving more tissue, as expected [47].

Regarding how this affects mechanical stresses on the remaining tooth structure, an *in vitro* study involving 72 maxillary first premolars compared Traditional Access Cavity (TradAC) with Conservative Access Cavity (ConsAC) was conducted. The samples were divided into four groups: the two different access cavity types were created, simulating class I caries in half of the samples and class II Black caries in the other half.

This was done to simulate real clinical situations as closely as possible, as only a small percentage of cases involve intact teeth. Specifically, to simulate class I caries, access was created exclusively at the occlusal level, while for class II caries, a mesial box was added. In ConsAC, this box remained independent of the occlusal access, whereas in TradAC, it was prepared continuously with the occlusal access, involving the complete removal of the pulp chamber roof according to traditional access guidelines [45].

In order to measure how these access cavities affected cuspal mechanical stresses, the intercuspal distance was measured after opening and completing the root canal treatment to assess cusp deflection. Maxillary premolars are most affected by this phenomenon. Subsequently, the teeth were subjected to a fracture test. From this measurement, it was found that increasing the dimensions of the endodontic cavity preparation maximized cusp deflection during restoration procedures.

However, within the limits of this study, cusp deflection was not significantly different in class I cavities between the TradAC and ConsAC groups, while it was indeed greater in the TradAC group compared to the ConsAC group in class II cavities.

To better understand why cusp deflection is so important for predicting the fracture resistance of endodontically treated teeth, an *in vitro* study involving 50 mandibular third molars was conducted. After the fracture test, the location of the fracture line on each sample was analyzed.

This analysis revealed that fractures occurred exclusively at two levels: on the floor of the pulp chamber or on the dental cusps. In teeth treated endodontically, almost all fractures occurred at the cuspal level, while they rarely involved the pulp chamber floor. In the control group consisting of intact teeth, fractures were more commonly located on the pulp chamber floor [48].

In another *in vitro* study, it was observed that the fracture pattern also varies between the two types of endodontic access (TradAC and ConsAC) following the fracture test. Samples treated with ConsAC more frequently exhibited mesiodistal fracture lines, whereas samples treated with TradAC showed a higher incidence of cuspal chipping patterns [47].

Furthermore, with the aim of replicating the clinical reality where many access cavities need to be created while taking into account dental structures already compromised by caries or previous restorations, in an *in vitro* study involving one hundred mandibular molars conducted by Ozyurek et al. [49], root canal treatments were simulated on class II caries. In this case, both ConsAC and TradAC were prepared as mesioocclusal class II endodontic cavities.

The result showed that, after confirming greater resistance in the control group, there was no statistically significant difference between the groups of teeth prepared with TradAC and ConsAC in class II treatments [49].

However, regarding the severity of fractures, specifically whether they were reparable or not, it emerged that the groups prepared with TradAC had a higher number of unreparable fractures. In particular, samples were restored in two different ways: in one, the proximal cavity was completely restored, and in the other, it was not.

From these findings, it can be deduced that the articles examined already cast doubt on the influence of different access cavities on the amount of mechanical stress subsequently applied to the remaining dental structure, especially its critical portion in determining postendodontic dental fractures. As for fracture resistance, the results obtained were highly inconsistent with each other, contrary to the initial studies supporting Clark et al.'s thesis [13].

This is the result of several systematic reviews, which concur in stating that the influence of minimally invasive endodontic preparations on tooth fracture resistance currently has limited supporting evidence.

In the systematic review conducted by Shabbir et al., the articles examined were categorized based on whether they focused on anterior or posterior teeth. Concerning anterior teeth, all the studies analyzed by the authors agreed that there was no relationship between different endodontic access cavities and the fracture resistance of the samples. However, for posterior teeth, the results were conflicting. In particular, 11 studies concluded that there was no relationship between access cavities and fracture resistance, while 9 studies demonstrated lower fracture resistance in TradAC compared with minimally invasive access cavities [13].

Conflicting results were also evident in the review by Silva et al. [26]. Among the 14 studies examined, 5 reported higher resistance in teeth prepared with minimally invasive access cavities compared with TradAC, while the remaining 9 studies did not observe a statistically significant difference between various endodontic access cavities [26].

Similar findings emerged in the review conducted by Saeed et al. [37], which aimed to compare TradAC and ConsAC. Out of a total of 10 studies, only 4 of them demonstrated greater fracture resistance in teeth prepared with ConsAC than in those prepared with TradAC. The remaining 6 studies reported no statistically significant differences in fracture resistance following the execution of TradAC or ConsAC for endodontic treatment [37].

Another systematic review, performed by Rover et al. [12], analyzed this parameter (fracture resistance) separately for different groups of teeth: incisors, upper premolars and molars, and lower premolars and molars.

Out of a total of 6 studies examined, only 2 demonstrated higher fracture resistance for ConsAC than for TradAC. Although it was noted that the final fracture resistance values exhibited considerable variability among the different studies, along with their standard deviations, the results obtained were diverse.

Specifically, in both upper and lower premolars, no statistically significant differences in fracture resistance were observed between samples prepared with TradAC and ConsAC. More specifically, for the lower premolars, there were no differences found between the prepared groups (whether TradAC or ConsAC) and the control groups consisting of intact teeth.

Regarding mandibular molars, elements prepared with ConsAC exhibited greater fracture resistance than those prepared with TradAC, whereas in upper molars, fracture resistance values were similar between the two access cavities.

Finally, concerning maxillary incisors, fracture resistance was similar across all three groups (TradAC, ConsAC, and the intact tooth group).

In the same systematic review, only one article addressing the ultraconservative access cavity (UltraAC) was included. It was noted that no statistically significant difference was observed between ConsAC, UltraAC, and intact teeth, while TradAC exhibited lower fracture resistance [12].

A discrepancy in results was also evident in the review conducted by Kapetanaki et al. [40], where significant result variability was noted among the 9 studies analyzed regarding fracture resistance. Among these, only 4 studies demonstrated higher resistance in teeth prepared with minimally invasive access compared with those prepared with TradAC, while the remaining 5 studies did not reveal statistically significant differences [40].

Focusing on truly minimally invasive access cavities (UltraAC and TrussAC), in an *in vitro* study, it was observed that teeth opened with UltraAC and TrussAC had significantly higher resistance (1712 N and 1805 N, respectively) compared to those with TradAC and ConsAC (702 N and 944 N, respectively). Simultaneously, the TrussAC and UltraAC groups did not exhibit significant differences between each other [44].

It is worth noting that this study exclusively involved mandibular molars since TrussAC can only be applied to diatoric elements. Therefore, it was deduced that achieving a better prognosis for endodontically treated teeth might be possible by minimizing dental tissue destruction during access, favoring the use of TrussAC or UltraAC.

In support of this, another study examined 60 molars, with half undergoing only a fracture test and the other half subjected to prior thermal cycling, involving 480 thermal cycles ranging from 5°C to 55°C in a dedicated thermal cycler before evaluating their fracture resistance.

It was observed that thermal cycling significantly reduced the fracture resistance of teeth prepared with both TradAC and TrussAC, with the lowest resistance seen in the TradAC group subjected to thermal cycling. On the other hand, teeth prepared with TrussAC did not exhibit significantly lower fracture resistance compared with the intact tooth control groups. This suggests that TrussAC could indeed represent a solution for improving the long-term prognosis of endodontically treated molars, where feasible [46].

Regarding UltraAC, in a study by Maske et al. [48], it was found that it did not provide any advantages in terms of fracture resistance compared to less conservative access cavities, especially ConsAC and TradAC [48].

An important mention should be made of another systematic review conducted by Mandil et al. [38], where one specific study demonstrated that ConsAC had a higher percentage of irreparable dental fractures due to fracture lines located more cervically in the tooth, resulting in greater loss of dental crown compared to other access cavities [38].

All authors, in light of the conflicting results, agree on highlighting some critical issues related to the methodology employed in the analyzed studies. Concerns have been raised about the selection of tooth samples for various study groups. It is believed that in many *in vitro* studies conducted so far, instead of prior analysis of both external and internal anatomy of the samples using CBCT, only the external tooth anatomy was observed for the purpose of distribution among the different groups, undermining the achievement of a more uniform distribution among them [26].

Another often overlooked factor, for the purpose of achieving a homogeneous distribution among the various study groups, is the age of the teeth, as aging is known to negatively affect dentin resistance [26].

In addition, it should be noted that not all studies perform, after the root canal procedure, the filling and coronal restoration of the sampled teeth [37].

Another critical aspect is the difficulty of replicating real oral clinical conditions in *in vitro* studies, particularly in reproducing intraoral masticatory forces in laboratory-based fracture tests [37].

In this regard, some authors emphasize that not all such studies simulate the presence of the periodontal ligament before subjecting the samples to the fracture test (7), and even when done, the techniques for simulating it vary significantly. In one of the analyzed studies, for instance, the apical ends of each tooth were covered with wax up to the amelocemental junction [46].

Further variations exist in the methodology of fracture resistance tests themselves: some authors applied a continuous compressive force to the central fossa at a 30° angle, while another used a 45° angle, and yet another used a 135° angle [12].

If we were to focus on the root canal treatment performed on the samples after their access opening, an additional variable arises from the techniques used for canal shaping and composite resin restoration.

Regarding canal shaping, it is logical to deduce that the more conservative the shaping is, the greater the preservation of the pericervical dentin, which is considered crucial in the philosophy of minimally invasive access openings, reducing the influence of the access cavities on fracture resistance. Conversely, it is reasonable to suggest that more aggressive instrumentation will maximize the criticality of the access cavity for the long-term prognosis of the dental element.

To illustrate this, it is useful to revisit the *in vitro* study conducted by Maske et al. [48], where both ConsAC and especially UltraAC did not show greater fracture resistance compared to TradAC. In this study, a very conservative shaping was performed without the execution of preflaring (preparation of the coronal third), resulting in greater preservation of pericervical dentin. Moreover, the instrumentation protocol used may also have an impact, particularly the type of endodontic instruments. In the aforementioned study by Maske et al. [48], the ProDesign Logic® continuous rotary system was employed, characterized by low taper and high flexibility, which makes it highly conservative.

It can be deduced that this, too, may have contributed to the lack of statistically significant differences among the various access openings in terms of mechanical resistance [48].

Finally, in the study by Ozyurek et al. [49], where not all teeth were restored after root canal treatment, it was noted that resin restoration indeed provides greater fracture resistance to the dental element compared to placing a single crown or an inlay. However, it was emphasized that this does not reflect the majority of clinical situations.

On the other hand, the fact that the majority of fractures occurring at the cuspal level seems to encourage resin restorations in more uncertain situations, at the expense of other prosthetic restorations [45, 47, 48]. Nevertheless, it has been observed that restoration procedures in cavities prepared with ConsAC are more challenging to perform [48].

Moreover, with regard to additional variables, they could be represented by the restorative materials and the methodology of their application. In the study by Ozyurek et al. [49], where restorations were carried out using two different resin composites, it was found that the teeth restored with SDR® exhibited greater resistance compared to EverX Posterior®. The latter is characterized by higher viscosity, which suggests that it may adapt less to the cavity walls, partially affecting the fracture resistance of the restored element.

For this purpose, according to another study cited by Ozyurek et al. [53], applying composite in oblique layers may yield better results than bulk application.

In order to determine how different access cavities affect the adaptation of the coronal restorative material, Silva et al. examined the formation of voids in the adhesive-composite interface (“gaps”) and within the composite restoration itself (“voids”) in an *in vitro* study, comparing the traditional access cavity with the ultraconservative technique [54].

Following the results of this study, which demonstrated a higher incidence of gaps in UltraAC compared to TradAC, with no statistically significant difference in the formation of internal voids within the restoration, it can be inferred that, while resin restoration remains the primary choice in MiniAC due to its cost-effectiveness, speed, good aesthetic performance, and less invasiveness compared to indirect restorations, these access cavities are more challenging to restore. The cavity size can make it difficult to apply incremental layering techniques, resulting in a higher risk of adhesive failures or gap formation at the interface between the restorative material and the cavity walls [54].

## 5. Conclusions

From this literature review regarding the advantages and disadvantages brought about by conservative and minimally invasive access cavities, it is evident that the results are still conflicting in various aspects.

These cavity designs, particularly conservative access (ConsAC), ultraconservative access (UltraAC, also known as “Ninja”), and the “Truss” access (TrussAC), have been proposed as alternatives to the traditional access (TradAC) with the aim of addressing the complication of dental fractures following endodontic treatment.

However, while it is true that reducing the size of the access cavity corresponds to less destruction of pericervical dentin and less mechanical stress on the cuspal level, as most fracture lines in endodontically treated teeth are localized at the level of dental cusps, the articles related to fracture resistance tests have provided highly discordant results. Specifically, for teeth in the anterior sectors, the results are not particularly optimistic, as the majority of studies do not find a relationship between different endodontic access cavities and the fracture resistance of specimens. In the posterior sector, on the other hand, there is more discrepancy, with most of the positive results for minimally invasive cavities seeming to primarily concern molars. The TrussAC itself can only be applied to these teeth.

It can be deduced, therefore, that currently, the influence of endodontic preparations on the fracture resistance of teeth has limited and insufficient supporting evidence.

Authors in this regard agree on the need to conduct a greater number of studies, highlighting how there are important methodological issues in the literature concerning the selection of tooth samples and the difficulty in replicating intraoral masticatory forces *in vitro*, as well as the presence of the periodontal ligament.

## Figures and Tables

**Table 1:** The main data extracted from the included articles are represented; specifically, all the conclusions of the systematic and narrative reviews of the literature relating to fracture resistance in relation to different cavity designs have been reported.

Authors	Data	Country	Type of study	Outcome	Study included
Shabbir et al. [36]	2021	USA	Review	Classification	49
*Conclusion: Minimally invasive access cavity designs present more risk than benefit on the outcome of endodontic treatment*					

Silva et al. [26]	2020	Brazil	Review	Classification	28
*Conclusion: There is a lack of supporting evidence for the introduction of minimally invasive access cavity preparation into routine clinical practice*					

Ballester et al. [27]	2021	France, Egypt, Malaysia	Systematic review and meta-analysis	Fracture resistance	33
*Conclusion: The evidence available does not support the application of TrussAC. UltraAC might be applied in limited occasions*					

Saeed et al. [37]	2021	United Arab Emirates	Systematic review	Fracture resistance	8
*Conclusion: There is insufficient evidence to decide whether ConsAC is more advantageous than TradAC in terms of fracture toughness*					
Silva et al. [12]	2018	Brazil	Systematic review	Fracture resistance	6
*Conclusion: There is no evidence that supports the use of ConsAC over TradAC for the increase of fracture resistance*					

Mandil et al. [38]	2022	Saudi Arabia	Review	Fracture resistance	\
*Conclusion: ConsAC, UltraAC, and TrussAC used as an alternative to TradAC access*					

Silva et al. [39]	2022	Brazil	Review	Fracture resistance	\
*Conclusions: There are no clear indications for the ConSAC and UltraAC openings that can guide the decision-making process*					

Kapetanaki et al. [40]	2021	Greece	Review	Tooth prognosis	22
*Conclusion: TradAC represent the safest method for preventing iatrogenic complications*					

Motiwala et al. [41]	2022	Pakistan	Systematic review and network meta-analysis	Fracture resistance	14
*Conclusion: ConsAC was the most favourable access cavity design when compared to others*					

Chan et al. [42]	2022	China	Review	Fracture resistance and tooth prognosis	44
*Conclusion: The currently available evidence is insufficient to support the use of UltraAC indiscriminately in routine endodontic practice*					

Maqbool et al. [43]	2020	Malaysia	Review	Fracture resistance and tooth prognosis	\
*Conclusion: Potential deleterious effects of such access cavity design rather than emphasizing the preservation of tooth structure alone*					

**Table 2:** The main data extracted from the ex vivo studies included are reported; furthermore, the main conclusions of the studies were reported.

Autor	Data	Country	Type of study	Number teeth\ roots	Objective	Access cavity	Conclusions
Patil et al. [44]	2022	India	Vitro, ex vivo	50	Fracture resistance	TradAC, ConsAC, UltraAC, TrussAC	TrussAC the fracture strength of teeth compared with the ConsAC
Mowlood et al. [45]	2022	Iraq	Vitro, ex vivo	62	Fracture resistance	ConsAC, TradAC	ConsAC could improve the resistance of the tooth to fracture
Saberi et al. [46]	2020	Iran	Vitro, ex vivo	60	Fracture resistance	TrussAC, TradAC	TrussAC increases fracture toughness
Shah et al. [47]	2018	India	Vitro, ex vivo	60	Fracture resistance	ConsAC, TradAC	Increased resistance to fracture in ConsAC
Maske et al. [48]	2021	Brazil	Vitro, ex vivo	50	Fracture resistance	TradAC, ConsAC	The type of access cavity preparation did not increase the fracture strength
Özyürek et al. [49]	2018	Turkey	Vitro, ex vivo	62	Fracture resistance	TradAC, ConsAC	ConsAC preparation did not increase the fracture strength of teeth
Barbosa et al. [50]	2020	Brazil	Vitro, ex vivo	30	Efficacy of canal instrumentation, microbial reduction and fracture resistance	TrussAC, TradAC, ConsAC	The ConsAC could improve the resistance of the tooth to fracture compared to TradAC
Rover et al. [51]	2020	Brazil	Vitro, ex vivo	40	Fracture resistance, shaping and filling ability and pulp chamber cleaning	ConsAC	ConsAC was associated with significantly more voids in root canal fillings
Plotino et al. [52]	2017	Italy	Vitro, ex vivo	160	Fracture resistance	TradAC, ConsAC, UltraAC	TradAC access showed lower fracture strength

## Data Availability

All data generated or analyzed during this study are included in this published article.

## Supplementary Materials

Supplementary Materials Supplemental Files. Narrative Review checklist.
