# Supplementary figures and images for: Influence of Cavity Designs on Fracture Resistance: Analysis of the Role of Different Access Techniques to the Endodontic Cavity in the Onset of Fractures: Narrative Review
Source: ScientificWorldJournal. 2024 Jul 30;2024:1648011. doi: 10.1155/2024/1648011 (PMC11303043; doi:10.1155/2024/1648011)

**Supplemental Files.** Narrative Review checklist


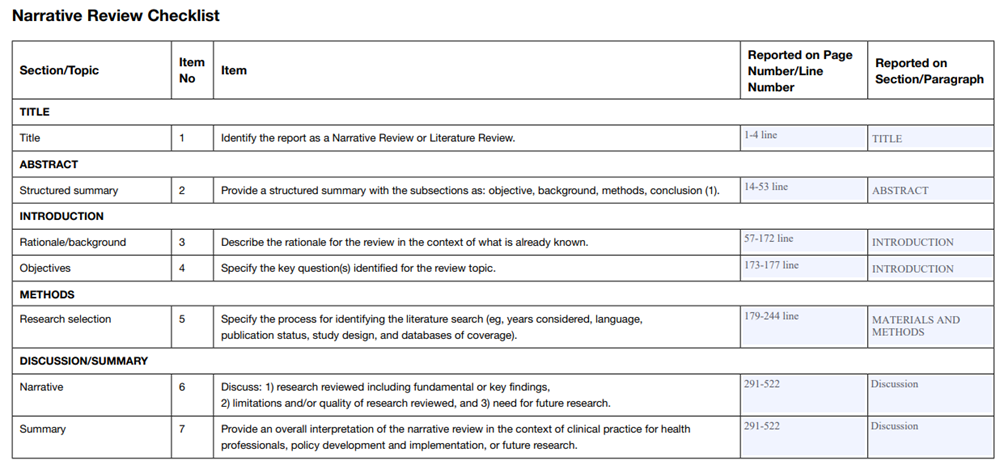

Supplement: Supplementary Materials — Supplemental Files. Narrative Review checklist. [file 1648011.f1.docx]
